# Aging exaggerates blood pressure response to ischemic rhythmic handgrip exercise in humans

**DOI:** 10.14814/phy2.15125

**Published:** 2021-11-24

**Authors:** Daisuke Hasegawa, Amane Hori, Yukiko Okamura, Reizo Baba, Kenichi Suijo, Masaki Mizuno, Jun Sugawara, Koji Kitatsuji, Hisayoshi Ogata, Kaoru Toda, Norio Hotta

**Affiliations:** ^1^ Graduate School of Life and Health Sciences Chubu University Kasugai Japan; ^2^ Nagoya Heisei College of Nursing and Medical Care Nagoya Japan; ^3^ Japan Society for the Promotion of Science Tokyo Japan; ^4^ College of Life and Health Sciences Chubu University Kasugai Japan; ^5^ Department of Applied Clinical Research University of Texas Southwestern Medical Center Dallas Texas USA; ^6^ Human Informatics and Interaction Research Institute National Institute of Advanced Industrial Science and Technology Tsukuba Japan

**Keywords:** acidosis, exercise pressor reflex, muscle mechanoreflex, muscle metaboreflex, postexercise muscle ischemia

## Abstract

Ischemic skeletal muscle conditions are known to augment exercise‐induced increases in blood pressure (BP). Aging is also a factor that enhances the pressor response to exercise. However, the effects of aging on the BP response to ischemic exercise remain unclear. We, therefore, tested the hypothesis that aging enhances the BP response to rhythmic handgrip (RHG) exercise during postexercise muscle ischemia (PEMI). We divided the normotensive participants without cardiovascular diseases into three age groups: young (*n* = 26; age, 18–28 years), middle‐aged (*n* = 23; age, 35–59 years), and older adults (*n* = 23; age, 60–80 years). The participants performed RHG exercise with minimal effort for 1 min after rest with and without PEMI, which was induced by inflating a cuff on the upper arm just before the isometric handgrip exercise ended; the intensity was 30% of maximal voluntary contraction force. Under PEMI, the increase in diastolic BP (DBP) from rest to RHG exercise in the older adult group (Δ13 ± 2 mmHg) was significantly higher than that in the young (Δ5 ± 2 mmHg) and middle‐aged groups (Δ6 ± 1 mmHg), despite there being no significant difference between the groups in the DBP response from rest to RHG exercise without PEMI. Importantly, based on multiple regression analysis, age remained a significant independent determinant of both the SBP and DBP responses to RHG exercise during PEMI (*p* < 0.01). These findings indicate that aging enhances the pressor response to ischemic rhythmic exercise.

## Graphical abstract

The systolic and diastolic blood pressure responses to low‐intensity rhythmic handgrip exercise with postexercise muscle ischemia in older adults were demonstrated to be higher than that of young, and young and middle‐aged groups, respectively. Additionally, aging persisted as a significant determinant explaining the variance in the blood pressure response to low‐intensity rhythmic handgrip exercise during postexercise muscle ischemia. These finding suggests that aging enhances pressor response to ischemic exercise.

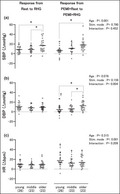

## 1 INTRODUCTION

During physical activity, the cardiovascular responses reflexively increase to match the systemic oxygen delivery with the metabolic demand of active skeletal muscles. The modulatory reflex mechanisms of the aorta and carotid arteries (arterial baroreflex) (Raven et al., 2019), neural activation originating from the higher motor center (central command) (Williamson et al., 2002), and reflex activity from active skeletal muscles (the exercise pressor reflex) (Smith et al., 2006) adjust the autonomic nerve system to regulate blood pressure (BP) during exercise. The exercise pressor reflex, activated by mechanical (muscle mechanoreflex) and metabolic stimuli (muscle metaboreflex) (Smith et al., 2006) of the working skeletal muscles, primarily and substantially contributes to an increase in BP in response to exercise (Smith et al., 2005, 2006).

The pressor response to systemic and local muscle exercise is known to be exaggerated by aging (Daida et al., 1996; Milia et al., 2015; Trinity et al., 2018). For example, Trinity et al. (2018) demonstrated that the increase in BP via the exercise pressor reflex to plantar flexion in older women is greater than that in younger women, considering that the age groups had equivalent metabolic responses in the working muscles. In addition, the pressor response to exercise is exaggerated when the skeletal muscles are in an acute ischemic or acidic condition (Cornett et al., 2000; Cristina‐Oliveira et al., 2020; Cui et al., 2008). For instance, Cornett et al. (2000) showed that the BP response to rhythmic handgrip (RHG) exercise was augmented by forearm circulatory arrest. Hence, it is evident that aging and ischemia are major factors that enhance the pressor response to exercise. However, the synergistic effect of these two factors, that is, whether ischemia further augments an aging‐induced exaggeration of pressor response to exercise, remains unclear.

An exaggerated pressor response is known to potentiate acute risks of adverse cardiovascular events during (Laukkanen et al., 2006) or after exercise (Laukkanen et al., 2004), even in healthy adults. Furthermore, previous evidence suggests that even in normotensive individuals, repetition in an elevated pressor response to physical activities chronically affects the heart and blood vessel, leading to an increased risk of future cardiovascular disease‐related death (Weiss et al., 2010). Thus, an exaggerated BP response to physical activities in daily life could strongly affect an individual's health and life expectancy. Hence, it is worthwhile to reveal effect of aging on BP response to ischemic exercise, because the older population is growing in many Asian and Western countries, and ischemic situations may occur not only while performing physical fitness exercises, but also during activities of daily life (e.g., ascending many flights of stairs or lifting heavy objects without taking breaks).

Therefore, the aim of this study was to investigate the effects of aging on cardiovascular response to dynamic exercise under ischemic muscle conditions. To accomplish this aim, we measured BP and heart rate (HR) response to RHG exercise during postexercise muscle ischemia (PEMI) (Alam & Smirk, 1937; Cornett et al., 2000; Cui et al., 2008). Although the resting BP linearly increases with advancing age (Kotchen et al., 1982), it is unclear whether BP responses to ischemic exercise and resting BP level would show a parallel change with advancing age. Nevertheless, nearly all studies investigating the effect of aging on pressor response to exercise only compared younger and older groups of participants (Markel et al., 2003; Milia et al., 2015; Sidhu et al., 2015; Trinity et al., 2018); few studies examined the influence of an advancing age on the BP response to exercise (Daida et al., 1996). Hence, the present study included three age groups: young, middle‐aged, and older adults. We hypothesized that an advancing age incrementally potentiated BP response to RHG exercise during PEMI.

## 2 METHODS

### 2.1 Sample size calculation and participants

A priori statistical power analysis was performed to determine the sample size needed for the present study using, G*power 3.1.9.7. We determined that a minimum sample size of 81 was needed to achieve a power (1 − *β*) of more than 0.80, which was required to reject the null hypothesis, with a medium effect size (*f* = 0.25) and an error probability of 0.05 (*α*), using a repeated measure two‐way analysis of variance (ANOVA) (number of groups = 3).

A total of 82 individuals with physically active lifestyles were recruited to participate in the present study. Among them, we excluded 10 who were considered to have hypertension (SBP > 140 mmHg or DBP > 90 mmHg) (Umemura et al., 2019) and included the remaining 72 normotensive individuals as the study participants. To evaluate the effects of aging, we divided the participants into three age groups based on a previous study (Zhang et al., 2020): young (*n* = 26, 14 men and 12 women, 18–34 years old), middle‐aged (*n* = 23, 11 men and 12 women, 35–59 years old), and older adults (*n* = 23, 9 men and 14 women, 60–80 years old). None of the participants had ever used antihypertensive medications. Moreover, none of the participants had diabetes (Kim et al., 2019), heart failure (Smith et al., 2006), kidney disease (Park & Middlekauff, 2015), peripheral arterial disease (Stone & Kaufman, 2015), or peripheral neuropathy (Grotle et al., 2017), which are known to affect the pressor responses to exercise. The characteristics of the groups are shown in Table 1.

**TABLE 1:** Participant characteristics

	Young (*n* = 26)	Middle‐aged (*n* = 23)	Older adult (*n* = 23)	*p*‐value
	Mean ± SE	Mean ± SE	Mean ± SE	
Age (years)	22 ± 1 (18–28)	47 ± 2 (35–59)	69 ± 1 (60–80)	<0.01^†^
Female/male ratio (%)	12/14 (46/54%)	12/11 (52/48%)	14/9 (61/39%)	0.59^χ^
Ratio of smoker (%)	5/26 (19%)	1/23 (4%)	4/23 (17%)	0.30^χ^
Height (cm)	166.4 ± 1.9	164.4 ± 1.8	157.9 ± 1.9	0.01*
Body mass (kg)	63.7 ± 2.2	64.1 ± 2.7	52.4 ± 1.8	<0.01^‡^
BMI (kg/m^2^)	22.9 ± 0.6	23.7 ± 0.9	20.9 ± 0.5	0.01^‡^
Body fat percentage (%)	23.3 ± 1.7	25.4 ± 1.9	22.4 ± 1.7	0.47
Muscle rate (%)	72.6 ± 1.6	70.5 ± 1.8	72.7 ± 1.8	0.61
CAVI	6.0 ± 0.1	7.0 ± 0.1	8.5 ± 0.2	<0.01^†^
MVC (N)	344 ± 22	316 ± 18	247 ± 17	<0.01^‡^

Abbreviations: BMI, body mass index; CAVI, cardio‐ankle vascular index; MVC, maximum voluntary contraction.

* *p* < 0.05 versus young.

^†^ *p* < 0.05, age‐group dependent.

^‡^ *p* < 0.05 versus young and middle‐aged groups.

^χ^ *p*‐value achieved using a chi‐square or Fisher's exact test.

From the day prior to conducting the experiments, the participants were asked to avoid alcohol, caffeine, and unusually long and/or high‐intensity training sessions. The participants did not consume any food for at least 2 h before the experiments. This study was approved by the Chubu University Ethics Committee (No. 290077‐3). All participants provided written informed consent.

### 2.2 Study design

To investigate the effect of aging on the cardiovascular response to ischemic exercise, we compared BP and HR responses to passive wrist movements (PWM) and RHG exercise during PEMI between the groups.

**FIGURE 1:**
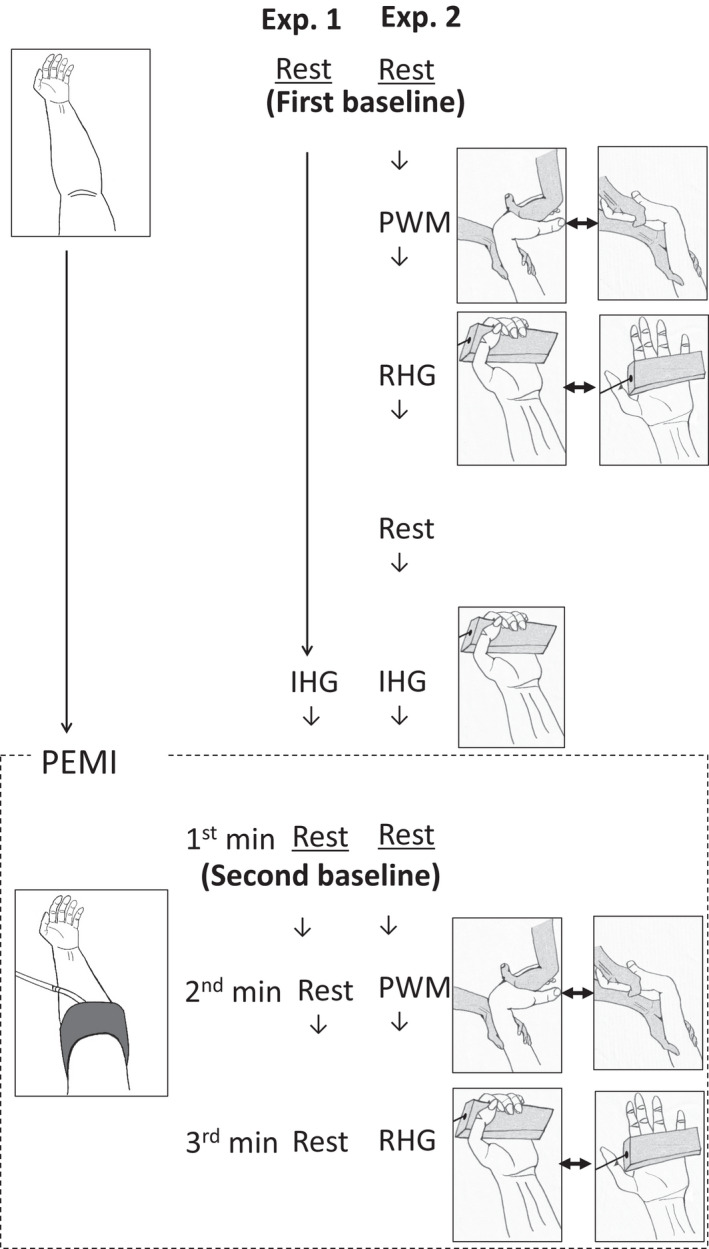
Protocols of experiments (Exp.) 1 and 2. In Exp. 1, after the baseline rest measurements (first baseline), the participants performed isometric handgrip (IHG) exercise, followed by a 3 min session of postexercise muscle ischemia (PEMI) without any exercise (rest). During IHG, the participants held the grip force transducer (shown in gray) in their left hand at 30% of their maximum voluntary contraction. PEMI was induced using the inflated cuff (shown in gray) on the left upper arm at a pressure of 250 mmHg. In Exp. 2, after the first baseline measurements, the participants performed passive wrist movement (PWM) and then rhythmic handgrip (RHG) exercise sessions, each for 1 min without PEMI. During PWM, the experimenter (shown in gray) fully extended and flexed the participant's left wrist (shown in white) at a rate of 60 repetitions/min. During RHG, the participant fully flexed and extended their left hand to grip the force transducer (shown in gray) at a rate of 60 repetitions/min using minimal effort. After a sufficient period of rest, the participants performed IHG exercise followed by a 3 min session of PEMI. Participants performed PWM during the 2nd minute (PEMI + PWM) and RHG exercise during the 3rd minute of the PEMI sessions (PEMI + RHG), after the 1st minute of rest time (PEMI + Rest). We defined the data measured during the 1st minute of PEMI as the second baseline

The present study comprised two experiments. Experiment (Exp.) 1 was performed to ensure that the participants’ BP and HR remained consistent throughout the 3 min period of PEMI (controlled rest during PEMI) (Figure 1). Exp. 2, the primary experiment, was performed to observe the BP and HR responses to 1 min of PWM, and subsequently, 1 min of RHG exercise after a 1‐min rest period during the 3 min period of PEMI (Figure 1). Finally, we also examined whether age remained a significant, independent determinant of pressor responses to ischemic exercise using a multivariate analysis, because some clinical parameters are known to affect cardiovascular response to exercise, for example, sex (Smith et al., 2019; Trinity et al., 2018), body mass index (BMI) (Limberg et al., 2018), and arterial stiffness (Thanassoulis et al., 2012).

### 2.3 Experimental protocols

Before the experimental procedures, participants were asked to sit on a comfortable chair for several minutes. In Exp. 1 (Figure 1), after the baseline rest measurements, participants performed isometric handgrip (IHG) exercise with their left arm (Figure 1), followed by a 3‐min session of PEMI at rest (Figure 1). In Exp. 2 (Figure 1), after the first baseline measurements, participants performed PWM (Figure 1), and subsequently, RHG exercise (Figure 1) for 1 min each, without PEMI. After a sufficient period of rest allowing the BP and HR to return to the first baseline values, the participants performed IHG exercise followed by a 3 min period of PEMI, similar to that in Exp. 1. PWM and RHG exercise were performed during the 2nd (PEMI + PWM) and the 3rd minute of PEMI (PEMI + RHG), respectively, after a 1 min rest period (PEMI + Rest) which we defined as the second baseline. This protocol was based on a previous study (Hotta et al., 2020). Exp. 1 and 2 were conducted on separate days for most participants. For some participants, Exp. 1 was performed on the same day, at least 1 h after completing Exp. 2, which allowed the BP and HR to return to the first baseline values of Exp. 2. Room temperature was maintained at approximately 25°C for both experiments.

### 2.4 IHG exercise and PEMI

The intensity of the IHG exercise was set at 30% of the individual's maximum voluntary contraction (MVC) force (Cui et al., 2008; Hotta et al., 2020; Park et al., 2008; Williamson et al., 2002), which was determined previously. Participants maintained their gripping force using a visual feedback system. The IHG exercise was performed for 2 min in the young and middle‐aged groups. However, this was reduced to 1 min for older adults owing to cardiovascular safety concerns (Hotta et al., 2020).

For PEMI, 5 s before the end of the IHG exercise, a Hokanson cuff (SC5, Hokanson, Inc.) was inflated using the Hokanson rapid cuff inflator (E20) and air source (AG101) on the left upper arm at a pressure of 250 mmHg.

### 2.5 PWM and RHG exercise

PWM and very low‐intensity RHG exercise with minimal force development were adopted to isolate the involvement of muscle mechanoreceptors from the central command and metaboreceptors (Park et al., 2008) and to minimize the engagement of muscle metaboreceptors (Batman et al., 1994) and the central command (Hotta et al., 2020), respectively.

For the PWM, the researcher fully extended and flexed the participant's left wrist at a rate of 60 repetitions/min, following the same method used in previous studies (Hotta et al., 2020; Park et al., 2008). For the RHG exercise, the participant fully flexed and extended their left hand at a rate of 60 repetitions/min using minimal effort (Hotta et al., 2020).

### 2.6 Measurements

#### 2.6.1 BP and HR

We measured systolic BP (SBP) and diastolic BP (DBP) on the participant's right upper arm during the latter 30 s of every minute using an oscillometric sphygmomanometer (Tango+, Sun Tech Medical Instruments Inc.). We assessed HR by recording an electrocardiogram (Tango+).

To compare the HR and BP responses to PEMI + PWM and PEMI + RHG between the groups, we used incremental (delta) values from the second baseline rest at the 1st minute of PEMI (PEMI + Rest; Figure 1), since the metabolic status elicited by IHG exercise could have differed between the groups (Markel et al., 2003).

#### 2.6.2 Rating of perceived exertion, blood lactate level, and arterial stiffness

The Borg scale (Borg, 1982) was utilized to obtain a rating of perceived exertion (RPE) for evaluating volitional effort sense immediately after finishing the RHG exercise without PEMI and PEMI + RHG sessions. We evaluated the lactate (La) level using a portable La measuring device (Lactate Pro2, Arkray). Blood samples were obtained from the participants’ fingertips at baseline and immediately after terminating the IHG exercise and PEMI + RHG session.

For the evaluation of arterial stiffness, we measured the cardio‐ankle vascular index (CAVI) using a semi‐automated vascular screening system (Vasera 1500 N, Fukuda Denshi, Japan) with the participant in the supine position after resting, as previously reported (Nishiwaki et al., 2015), based on the previously reported rationale (Namba et al., 2019). The mean value of each ankle's CAVI was used for analysis.

#### 2.6.3 Body composition

Whole body skeletal muscle and fat mass were assessed using a body composition analyzer based on multi‐frequency bioelectrical impedance analysis (BIA) (MC‐980, Tanita). The principle and reliability of multi‐frequency BIA have been described in previous reports (Heymsfield et al., 2015). We calculated both the skeletal muscle and fat mass percentages.

### 2.7 Statistical analysis

We first performed the Shapiro–Wilk test to confirm data normality. For comparisons between groups (young, middle‐aged, and older adults), one‐way ANOVA or the Kruskal–Wallis test was used. Furthermore, we performed two‐way ANOVA with repeated measures. The factors included “age” (young, middle‐aged, and older adults) and “stimulation mode” (Rest, PWM, RHG, IHG, PEMI, PEMI + Rest, PEMI + PWM, and PEMI + RHG). We selected the following tests for post hoc analysis: the Tukey or Steel‐Dwass test for fixed variable comparison, that is, “age,” and Shaffer's modified sequentially rejective Bonferroni procedure for the comparison of factors that were repeatedly measured, that is, “stimulation mode.” A chi‐square test or Fisher's exact test was used for the ratio comparisons.

We assessed the relationships between BP responses and clinical variables using Pearson's or Spearman's correlation. If a significant correlation was detected, a forward‐backward stepwise multiple regression analysis was used to identify significant determinants explaining the pressor response to ischemic exercise.

Data are expressed as mean ± standard error (SE). Statistical analyses were performed using R‐4.0.2, GraphPad Prism 8, and SPSS 25.0 software programs. The level of significance was set at *p* < 0.05.

## 3 RESULTS

The characteristics of the participants are summarized in Table 1. The height and body weight of the older adult group were significantly lower than that of the young group, and that of the young and middle‐aged group, respectively. However, the estimated total skeletal muscle and fat mass percentages were not significantly different between the groups. The CAVI increased significantly in an age‐group‐dependent manner. The MVC force in the older adult group was significantly lower than that in the young and middle‐aged groups. Accordingly, the mean grip force during IHG exercise in older adults (70 ± 5 N) was also significantly lower than that in the young (97 ± 6 N) and middle‐aged groups (91 ± 5 N). However, the relative values of MVC were not significantly (*p* = 0.11) different between the groups (28% ± 0% in the young, 29% ± 0% in the middle‐aged, and 28 ± 0% in the older adult group).

Thirty‐nine participants participated in Exp. 1. Table 2 shows the BP and HR responses to the IHG exercise and the 3 min period of PEMI. PEMI significantly increased BP as compared to rest, and BP did not significantly change during 3 min of PEMI in any of the age groups. The HR response to PEMI differed from the BP response. PEMI significantly increased HR as compared to rest, only at the 3rd minute regardless of the group, and HR at the 2nd and 3rd minute of PEMI significantly increased as compared to the 1st minute of PEMI in the older adult group.

**TABLE 2:** Circulatory responses to isometric handgrip (IHG) exercise followed by 3 min of postexercise muscle ischemia (PEMI) in Experiment 1

		*n*	Rest	IHG	PEMI			Rec	*p*‐value		
					1st min	2nd min	3rd min		Age	Stim. mode	Interaction
SBP (mmHg)	Young	12	114 ± 2	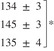	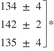	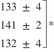	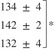	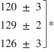	0.099	<0.001	0.126
	Middle‐aged	13	123 ± 3								
	Older adult	14	121 ± 3								
DBP (mmHg)	Young	12	71 ± 2	91 ± 3*	85 ± 3*	87 ± 3*	87 ± 3*	75 ± 3	0.011	<0.001	0.003
	Middle‐aged	13	80 ± 1^Y^	96 ± 2*	94 ± 3*	94 ± 3*	94 ± 2*	88 ± 2*^Y^			
	Older adult	14	80 ± 1^Y^	88 ± 2*^M^	86 ± 1*	88 ± 2*	88 ± 2*	83 ± 2			
HR (bpm)	Young	12	61 ± 2	78 ± 3*	67 ± 3^†^	67 ± 2^†^	69 ± 2*	64 ± 3	0.64	<0.001	0.016
	Middle‐aged	13	61 ± 1	72 ± 2*	63 ± 2^†^	64 ± 2^†^	66 ± 2*^†^	64 ± 2*			
	Older adult	14	65 ± 2	71 ± 2*	65 ± 2^†^	66 ± 2*^‡†^	68 ± 2*^‡†^	66 ± 2			

Note

The young and middle‐aged groups performed 2 min of IHG exercise whereas the older adult group completed it for 1 min. Thus, we adopted the value at the 2nd minute of IHG exercises in both the young and middle‐aged groups and the value at the 1st minute of it in the older adult group. SBP, systolic blood pressure; DBP, diastolic blood pressure; HR, heart rate; Rec, recovery. Due to there being no significant interaction in SBP, time series data (the effect of stimulation mode) were analyzed regardless of the group. *p < 0.05 versus Rest; ^†^ *p* < 0.05 versus IHG; ^‡^ *p* < 0.05 versus PEMI at 1st minute; ^Y^ *p* < 0.05 versus young group; ^M^ *p* < 0.05 versus middle‐aged group.

Table 3 shows the changes in SBP, DBP, and HR obtained from Exp. 2. BP and HR significantly and gradually increased in response to PWM followed by RHG exercise without PEMI as compared to the first baseline rest in all the age groups, except for the DBP response of the middle‐aged group. Under the influence of PEMI, BP was significantly elevated exclusively during PEMI + RHG, but not during PEMI + PWM, as compared to the second baseline rest (PEMI + Rest). In terms of the group comparison of SBP, even though the first and second baseline rest periods were not significantly different between the groups, the values obtained during RHG exercise without and with PEMI in the older adult group were significantly higher than those obtained in the young group. Regarding DBP, no significant difference was detected in PEMI + Rest between the young and older adult groups. However, DBP during PEMI + RHG in the older adult group was significantly higher than that in the young group. Conversely, HR during the IHG exercise, PEMI + Rest, and PEMI + RHG in the middle‐aged and older adult groups was significantly lower than those in the young group.

**TABLE 3:** Circulatory responses to isometric handgrip (IHG) exercise and rest, passive wrist movement (PWM), and rhythmic handgrip (RHG) exercise without and with postexercise muscle ischemia (PEMI)

		n	Rest	PWM	RHG	IHG	PEMI			Rec	*p*‐value		
							Rest	PWM	RHG		Age	Stim. mode	Interaction
SBP (mmHg)	Young	26	116 ± 2	120 ± 2*	124 ± 2*^†^	136 ± 3*	135 ± 3*	137 ± 3*	142 ± 3*^⁑‡^	126 ± 2*	0.232	<0.001	0.004
	Middle‐aged	23	119 ± 2	123 ± 2*	128 ± 2*^†^	142 ± 3*	138 ± 3*	139 ± 3*	149 ± 3*^⁑‡^	129 ± 3*			
	Older adult	23	118 ± 2	124 ± 3*	135 ± 4*^†Y^	138 ± 4*	139 ± 4*	139 ± 4*	157 ± 5*^⁑‡Y^	135 ± 4*			
DBP (mmHg)	Young	26	68 ± 2	75 ± 2*	79 ± 2*^†^	89 ± 2*	86 ± 2*	87 ± 1*	91 ± 2*^⁑‡^	79 ± 2*	0.001	<0.001	<0.001
	Middle‐aged	23	79 ± 1^Y^	83 ± 2^Y^	87 ± 2*^Y^	95 ± 2*	92 ± 2*^Y^	92 ± 2*	98 ± 2*^⁑‡Y^	89 ± 2*^Y^			
	Older adult	23	79 ± 2^Y^	84 ± 2*^Y^	88 ± 2*^†Y^	89 ± 2*	88 ± 2*	89 ± 2*	101 ± 2*^⁑‡Y^	86 ± 2*^Y^			
HR (bpm)	Young	26	66 ± 2	69 ± 2*	76 ± 2*^†^	84 ± 3*	74 ± 2*	79 ± 2*^⁑^	91 ± 3*^⁑‡^	71 ± 2*	0.031	<0.001	<0.001
	Middle‐aged	23	64 ± 1	66 ± 1*	73 ± 2*^†^	75 ± 2*^Y^	67 ± 1*^Y^	71 ± 2*^⁑^	80 ± 2*^⁑‡Y^	67 ± 1*			
	Older adult	23	64 ± 2	65 ± 2	74 ± 3*^†^	71 ± 2*^Y^	66 ± 2*^Y^	69 ± 2*^Y^	80 ± 3*^⁑‡Y^	67 ± 2*			

Note

The young and middle‐aged groups performed 2 min of IHG exercise whereas the older adult group completed it for 1 min. Thus, we adopted the value at the 2nd minute of IHG exercises in both the young and middle‐aged groups and the value at the 1st minute of it in the older adult group. SBP, systolic blood pressure; DBP, diastolic blood pressure; HR, heart rate; Rec, recovery. *p < 0.05 versus Rest; ^⁑^ *p* < 0.05 PEMI + Rest versus PEMI + PWM or PEMI + RHG; ^†^ *p* < 0.05 PWM versus RHG; ^‡^ *p* < 0.05 PEMI + PWM versus PEMI + RHG; ^Y^ *p* < 0.05 versus young group.

**FIGURE 2:**
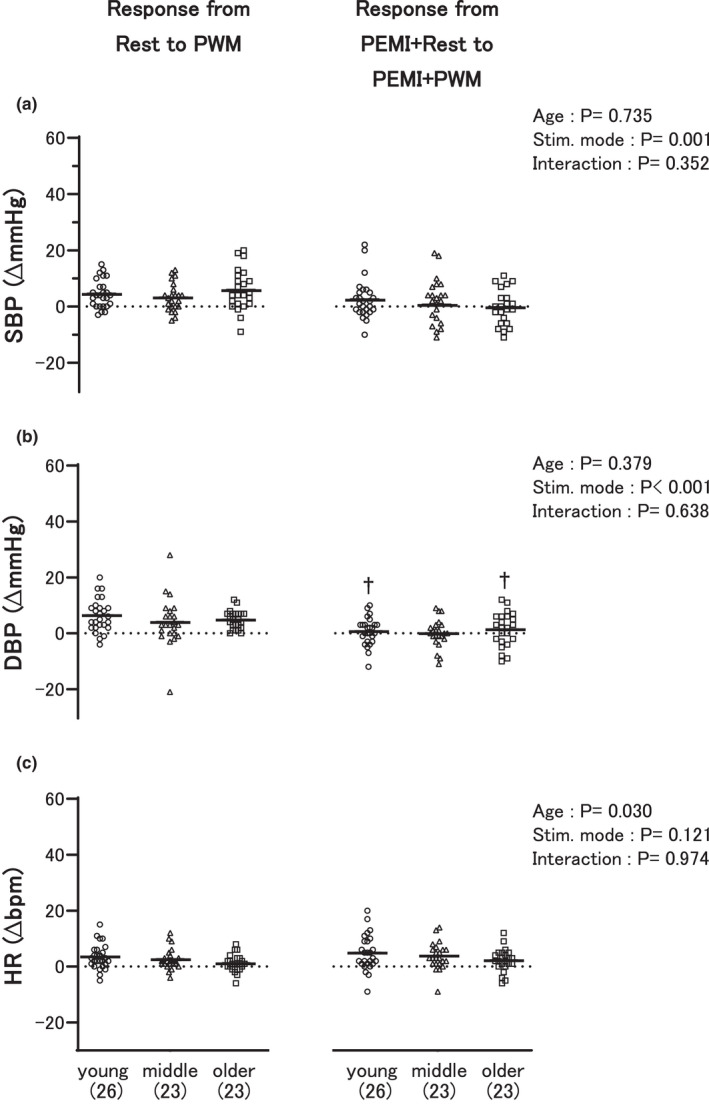
Comparisons of the circulatory responses between the three age groups from the first baseline rest to passive wrist movement (PWM) (left) and from the second baseline rest (PEMI + Rest) to PWM (PEMI + PWM) during postexercise muscle ischemia (PEMI) (right). The first and second baselines are explained in Figure 1. SBP (a), DBP (b), and HR (c) indicate systolic and diastolic blood pressure, and heart rate, respectively. “Young,” “middle,” and “older” represent the young, middle‐aged, and older adult groups, respectively. ^†^ *p* < 0.05 versus PWM in each group. The number of participants is shown in parentheses. The dots and bars illustrate individual and mean values, respectively

The left side of Figure 2 shows the circulatory responses to PWM from the first baseline rest, whereas the right side of Figure 2 shows the changes in the measurements from the second baseline (PEMI + Rest). No significant difference in response was detected between the groups to either PWM without PEMI or PEMI + PWM.

To evaluate the volitional effort sense during the RHG exercise, we measured the RPE; however, we failed to measure it for each participant in the young (i.e., *n* = 25) and middle‐aged (i.e., *n* = 22) groups. During the RHG exercise without PEMI, the RPE did not differ between the groups (*p* = 0.71). The RPE values in the young, middle‐aged, and older adult groups were 11.2 ± 0.4, 11.6 ± 0.3, and 11.6 ± 0.3, respectively. The RPE during the RHG exercise was significantly augmented by PEMI, regardless of the group (*p* < 0.01). The increment in RPE between the RHG exercise without PEMI and the PEMI + RHG in the older adult group (Δ3.1 ± 0.4) was significantly (*p* < 0.001) smaller than that in the young (Δ6.0 ± 0.5) and middle‐aged groups (Δ5.0 ± 0.4). The mean absolute force and %MVC during RHG exercise without and with PEMI were 22 ± 3 N (7% ± 1%) and 16 ± 3 N (5% ± 1%) in the young group, 17 ± 2 (5 ± 0%) and 13 ± 1 N (4 ± 0%) in the middle‐aged group, and 15 ± 2 N (6 ± 1%) and 15 ± 2 N (6 ± 1%) in older adult group, respectively. Age had no significant effect (*p* > 0.22) on either the absolute or relative values.

**FIGURE 3:**
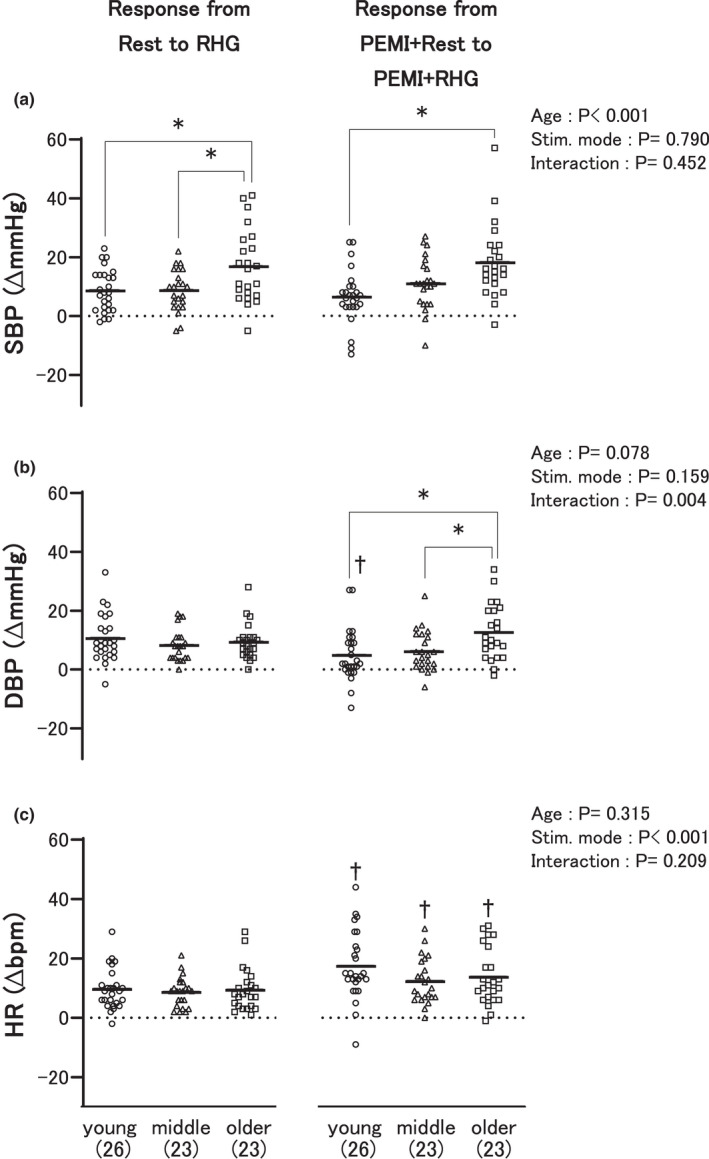
Comparisons of the circulatory responses between the three age groups from the first baseline rest to rhythmic handgrip (RHG) exercise (left) and from the second baseline rest (PEMI + Rest) to RHG exercise (PEMI + RHG) during postexercise muscle ischemia (PEMI) (right). The first and second baselines are explained in Figure 1. SBP (a), DBP (b), and HR (c) indicate systolic and diastolic blood pressure, and heart rate, respectively. “Young,” “middle,” and “older” represent the young, middle‐aged, and older adult groups, respectively. *p < 0.05; ^†^ *p* < 0.05 versus RHG in each group. The number of participants is shown in parentheses. The dots and bars illustrate individual and mean values, respectively

Figure 3 illustrates the most important result found in the present study, and demonstrates the SBP, DBP, and HR responses to RHG exercise similarly to Figure 2. The increment in SBP in response to RHG exercise without PEMI from the first baseline rest to the RHG exercise in the older adult group was significantly greater than that in the young and middle‐aged groups (Figure 3a). Similarly, the increment in SBP from the second baseline (PEMI + Rest) to PEMI + RHG in the older adult group was significantly higher than that in the young group. Noticeably, the increment in DBP from PEMI + Rest to PEMI + RHG in the older adult group was significantly higher than that in the young and middle‐aged groups, despite there being no significant difference between the groups in the DBP response to RHG exercise without PEMI (Figure 3b).

To estimate the degree of tissue acidosis, we measured the blood La levels of nine participants in the young group, eight in the middle‐aged group, and six in the older adult group. No significant differences between the groups were detected at rest (*p* = 0.83) (1.1 ± 0.1 mmol/L in the young, 1.1 ± 0.1 mmol/L in the middle‐aged, and 1.1 ± 0.1 mmol/L in the older adult group). IHG exercise significantly (*p* < 0.001) increased the blood La (2.7 ± 0.4 mmol/L, 2.5 ± 0.4 mmol/L, and 1.7 ± 0.3 mmol/L in the young, middle‐aged, and older adult groups, respectively) and PEMI + RHG further increased it significantly (*p* < 0.001; 3.7 ± 0.5 mmol/L, 3.8 ± 0.8 mmol/L, and 3.3 ± 0.4 mmol/L in the young, middle‐aged, and older adult groups, respectively). However, the changes in blood La were not significantly different between the groups because no significant interaction (*p* = 0.69) or effects of age (*p* = 0.53) were detected.

Table 4 shows single correlations between BP responses to PWM and RHG exercise and other parameters. Age was significantly correlated with the SBP response to RHG exercise and PEMI + RHG and the DBP response to PEMI + RHG. The SBP response to RHG exercise as well as the SBP and DBP responses to PEMI + RHG were significantly associated with the CAVI. In addition, DBP at rest, sex, and the MVC were significantly associated with the SBP response to RHG exercise and PEMI + PWM, and the DBP response to PWM and PEMI + RHG. No significant correlation was detected between smoking and any BP responses to PWM or RHG exercise with or without PEMI.

**TABLE 4:** Results of single correlation analysis between systolic (SBP) and diastolic blood pressure (DBP) responses to passive wrist movement (PWM) and rhythmic handgrip (RHG) exercise without and with postexercise muscle ischemia (PEMI) and clinical variables

Variables		Mean	SE	SBP (Δ mmHg)								DBP (Δ mmHg)							
				Response from Rest				Response from PEMI + Rest				Response from Rest				Response from PEMI + Rest			
				PWM		RHG		PEMI + PWM		PEMI + RHG		PWM		RHG		PEMI + PWM		PEMI + RHG	
				*r*	*p*	*r*	*p*	*r*	*p*	*r*	*p*	*r*	*p*	*r*	*p*	*r*	*p*	*r*	*p*
Age	years	45	2	0.10	0.41	0.36	**<0.01**	−0.08	0.49	0.41	**<0.01**	−0.12	0.32	−0.02	0.88	0.05	0.69	0.34	**<0.01**
SBP Rest	mmHg	118	1	0.05	0.66	0.19	0.12	−0.01	0.93	0.10	0.39	0.24	**0.04**	0.27	**0.02**	0.04	0.74	0.20	0.09
DBP Rest	mmHg	75	1	0.15	0.22	0.31	**<0.01**	−0.01	0.96	0.23	0.05	−0.12	0.34	−0.05	0.68	−0.03	0.77	0.10	0.41
Sex	Female = 0 (52.8%), male = 1 (47.2%)			0.20	0.09	0.09	0.47	0.26	**0.03**	0.07	0.56	0.26	**0.03**	0.12	0.31	−0.04	0.75	0.00	0.97
Smoking	Non‐smoker = 0 (86.1%), smoker = 1 (13.9%)			0.07	0.58	0.05	0.68	0.11	0.35	0.08	0.50	0.09	0.47	0.18	0.13	−0.08	0.53	0.01	0.96
BMI	kg/m^2^	22.5	0.4	−0.03	0.83	0.02	0.87	0.19	0.10	−0.06	0.62	0.02	0.88	−0.04	0.73	−0.01	0.91	−0.18	0.13
MVC	N	304	12	−0.02	0.87	−0.07	0.55	0.24	**0.04**	−0.04	0.74	0.24	**0.04**	0.02	0.90	−0.13	0.27	−0.29	**0.01**
CAVI		7.1	0.1	0.09	0.44	0.27	**0.02**	−0.06	0.62	0.34	**<0.01**	−0.10	0.40	−0.03	0.79	−0.04	0.73	0.34	**<0.01**
RPE PEMI + RHG	Δ	4.7	0.3	−0.13	0.30	−0.11	0.35	0.14	0.24	−0.11	0.38	0.06	0.65	−0.08	0.53	0.08	0.52	−0.17	0.17
HR Rest	bpm	65	1	−0.02	0.87	0.12	0.32	0.04	0.71	0.01	0.92	0.03	0.83	0.00	0.98	0.18	0.13	0.15	0.21
Lactate PEMI + RHG	Δmmol/L	2.5	0.3	−0.07	0.74	−0.23	0.29	0.08	0.72	0.19	0.40	0.09	0.68	−0.12	0.60	−0.04	0.86	0.05	0.81

Overall *p*‐values were assessed using the Spearman’s or Pearson’s correlation coefficient, depending on the data distribution. Bold‐faced values demonstrate a significant difference.

Abbreviations: BMI, body mass index; CAVI, cardio‐ankle vascular index; HR, heart rate; MVC, maximal handgrip strength; RPE, rating of perceived exertion.

We used the parameters that were significantly correlated with the BP response to exercise as candidates for independent variables in the multiple regression analyses. Importantly, the multiple regression analysis showed that age was a significant independent determinant of the SBP response to RHG exercise and PEMI + RHG and the DBP response to PEMI + RHG (Table 5).

**TABLE 5:** Summary of multiple regression analysis

Variables included in the model	*β*	*R* ^2^	*p*‐value
SBP response			
*to RHG*			
Age	0.389	0.140	0.001
DBP Rest	—	—	N.S.
CAVI	—	—	N.S.
to PEMI + PWM			
Sex	—	—	N.S.
MVC	—	—	N.S.
to PEMI + RHG			
Age	0.416	0.161	<0.001
CAVI	—	—	N.S.
DBP response			
to PWM			
Sex	0.272	0.061	0.021
SBP Rest	—	—	N.S.
MVC	—	—	N.S.
to RHG			
SBP Rest	0.334	0.099	0.004
to PEMI + RHG			
Age	0.359	0.117	0.002
MVC	—	—	N.S.
CAVI	—	—	N.S

Note

The independent variables that significantly correlated with systolic (SBP) and diastolic blood pressure (DBP) responses to passive wrist movement (PWM) or rhythmic handgrip (RHG) exercise without and with postexercise muscle ischemia (PEMI) in Table 4 were selected for these analyses. *β* and *R* ^2^ represent the standardized regression coefficient and a measure for the model prediction, respectively. The variance inflation factors of all the independent factors were <5. CAVI, cardio‐ankle vascular index; SBP and DBP Rest, SBP and DBP measured at rest; N.S., not significant.

## 4 DISCUSSION

The major findings from the present study are as follows. First, we confirmed that SBP and DBP did not significantly change during 3 min of PEMI. Second, the SBP response to RHG exercise without PEMI in the older adult group was significantly higher than that in the young and middle‐aged groups. This result is in accordance with previous studies (Daida et al., 1996; Milia et al., 2015; Trinity et al., 2018). Likewise, the SBP response to PEMI + RHG in the older adult group was significantly higher than that in the young group. Third, and most importantly, the DBP response to RHG exercise during PEMI in the older adult group was significantly higher than that in the young and middle‐aged groups, despite the DBP response to RHG exercise without PEMI not being significantly different between the groups. Fourth, these findings were supported by the result of the multiple regression analysis, which indicated that aging was a significant, independent enhancing factor for BP response to PEMI + RHG. Therefore, the present study is the first to show that aging increases the pressor response to ischemic rhythmic exercise, although BP response was not exaggerated clearly in proportion to age.

### 4.1 Potential mechanisms that may contribute to the augmented DBP response to ischemic exercise in older adults

Although the present study could not elucidate the mechanisms causing the ischemia‐revealed effect of aging on DBP response to exercise, several potential mechanisms can be proposed.

Trinity et al. (2018) demonstrated that aging augments the pressor response to exercise when the metabolic response in the working muscles is the same between different age groups, implying the possibility that aging increases the sensitivity and/or activation (gain) of the exercise pressor reflex. In the present study, we did not evaluate the metabolic perturbations elicited by exercise. It was assumed that the metabolic disturbances during exercise in the older adult group would diminish or be equivalent to those of the young and middle‐aged groups because: (1) herein, the duration of the IHG exercise in the older adult group was shorter than that in the young and middle‐aged groups; (2) aging skeletal muscles have a higher oxidative capacity (Miljkovic et al., 2015), and (3) the blood La response was not statistically different between the groups; however, La is not the only metabolite that stimulate the exercise pressor reflex and the sample sizes of La in all three groups were small. Nevertheless, the present study demonstrated that ischemia potentiated the aging‐related increase of DBP response to exercise. Previous animal and human studies have suggested that acute acidosis and/or a few metabolites increase the muscle mechanoreflex (Adreani & Kaufman, 1998; Cui et al., 2008; Hotta et al., 2019); however, the mechanism for this remains unclear. Hence, we speculate that aging exaggerates the acid‐induced increase in the muscle mechanoreflex, which may lead to aa greater DBP response to ischemic exercise in older adults.

It is generally accepted that the extent of the central command involvement is associated with volitional effort sense during exercise (Williamson et al., 2002). The present study showed that, although the RPE response to ischemic exercise was the smallest in the older adult group as compared to the other age groups, the DBP response to ischemic exercise was the greatest in the older adult group. Therefore, it is reasonable to assume that the difference in the sense of effort associated with the central command between the age groups did not have a major influence on the pressor response. However, further research is required because we did not directly evaluate the engagements of the central command, and because the RPE might be affected by age (Morishita et al., 2013).

In healthy young adults, the carotid baroreflex has been reported to attenuate the muscle metaboreflex‐induced pressor response (Ichinose et al., 2017). Conversely, aging has been suggested to decrease baroreflex function (Carrington & White, 2002). One of the reasons for the impaired function is age‐related vascular stiffening (Lipman et al., 2002). As the CAVI increased in an age‐group‐dependent manner, it is plausible, although speculative, that age‐related stiffening of the arterial wall might have resulted in a blurred baroreflex, which in turn might have induced the exaggerated DBP response to ischemic exercise observed in older adults.

It is reasonable to wonder why the synergistic effect of aging and ischemia was observed only for the DBP response, although it is not unusual that SBP and DBP responses to exercise are not altered parallelly (Brett et al., 2000). First, as compared to SBP, DBP is more influenced by changes in peripheral vascular resistance (Rowell, 1993). Previous evidence suggests that peripheral vasomotor activation is an important mechanism for determining the exercise pressor reflex in older adults (Sidhu et al., 2015). Thus, we believe that ischemia‐augmented aging‐induced peripheral vasomotor activation, resulting in the significant enhancement of DBP response to ischemic exercise in older adults in the present study. Further research involving stroke volume and blood flow measurement to calculate the cardiac output and peripheral resistance is required to validate our theory. Second, the SBP response to exercise without PEMI was not significantly different than that of exercise with PEMI in all age groups, indicating that the ischemic and/or exercise duration or intensity was insufficient to further augment an age‐related increase in SBP response. Third, we measured BP every 1 min and SBP was evaluated earlier than DBP in the present study; thus, the SBP may have been evaluated before the continuous hemodynamic alterations evoked by the effect of aging and ischemic exercise was reflected in SBP, leading to underestimation of the between‐group differences in SBP responses. Future studies using beat‐to‐beat monitoring systems to measure BP continuously are warranted to clarify this technical issue.

### 4.2 Methodological considerations for IHG exercise, PWM, and RHG exercise

It can be questioned whether a 1 min period of IHG exercise in the older adult group was enough to evoke significant metabolite production. Some previous studies have shown that PEMI followed by a 1 min period of IHG exercise induced the muscle metaboreflex (Hotta et al., 2020; Markel et al., 2003). We also confirmed that BP was upregulated by PEMI as compared to rest in the older adult group and that this upregulation was not significantly different between the groups in the present study. Therefore, it is evident that a 1 min period of IHG exercise was enough to produce significant metabolites during PEMI.

In terms of the shorter duration of IHG exercise in the older adult group, in this study, the BP and HR responses during and after IHG exercise in the older adult group were underestimated as compared to those in the young and middle‐aged groups. However, this may not be avoidable, even if the duration of IHG exercise was the same between the groups, because aging causes the skeletal muscle fibers to change to the oxidative fiber type (Miljkovic et al., 2015); thus, fewer metabolites stimulate thin‐fiber muscle afferents during exercise (Markel et al., 2003; Trinity et al., 2018). Despite this, the DBP response to ischemic exercise was significantly enhanced by aging, suggesting that the effect of aging on this response to ischemic exercise is powerful. However, the difference in the duration of IHG exercise did not allow us to directly compare the pressor response between the groups. Future studies involving the same duration of IHG exercise for all age groups are, thus, warranted.

In the present study, we attempted to isolate the effect of the muscle mechanoreflex on the pressor response by utilizing PWM (Park et al., 2008). The pressor response occurred during PWM; however, no significant difference was observed in the pressor response to ischemic PWM between the groups. Accordingly, the mechanism underlying the ischemia‐enhanced aging‐induced augmentation in DBP response to exercise could not be explained based on the between‐group differences in the muscle mechanoreflex. On the other hand, a significant age‐related increase in DBP response was observed during very low‐intensity ischemic RHG exercise performed with minimal effort to minimize the effects of the central command (Hotta et al., 2020) and additional muscle metaboreflex (Batman et al., 1994). This may have been due to technical differences in the PWM and RHG exercise; the RHG exercise‐activation of the muscle mechanoreflex is greater than that of PWM because PWM was not an exercise, but just a movement. However, the effects of central command and the muscle metaboreflex on the ischemia‐augmented the influence of aging on the pressor response to RHG exercise could not be ignored. In addition, we did not evaluate the between‐group differences in the baroreflex function changed during ischemic exercise. Accordingly, the present protocol could not conclude which neural factor played a key role in the observed response.

### 4.3 Limitations and strengths

We did not examine any influences of sex on the effect of aging on the pressor response to ischemic exercise in the present study due to the following reasons: (1) inadequate statistical power for comparing two (female and male) times three (young, middle‐aged, older adults) groups (total six groups); and (2) we did not gather information regarding the menstrual cycle and menopausal status of the female participants. However, it is well known that female hormones have an influence on the exercise pressor reflex (Smith et al., 2019). In fact, the sex‐specific impact of aging on the BP response to exercise has been reported (Trinity et al., 2018). Further research is needed in the future.

We also acknowledge the following as limitations. First, we did not assess the degree of pain that affects BP during PEMI. However, evidence suggests that afferent input signals elicited by noxious stimuli decrease with age (Taguchi et al., 2010). Thus, it cannot be presumed that the BP response to ischemic exercise in the older adult group was greater than those of the middle‐aged and young groups as a result of the greater pain experienced by the older adult participants. Second, the present study did not evaluate daily physical activity levels. Because the cardiopulmonary fitness level is suggested to be linked to the exercise pressor reflex (Mizuno et al., 2015), we cannot exclude the possibility that a greater pressor response to ischemic exercise in the older adult group was attributed to a lower level of daily physical activity. The skeletal muscle and fat mass percentages evaluated using BIA were not significantly different between the groups; however, BIA is affected by acute fluid consumption (Dixon et al., 2009), and we did not control the participants’ water intake before BIA measurement. In addition, all participants led physically active lives. In fact, the mean BMI in the older adult group was lower than that of the older adult population (Tarui et al., 2020). Therefore, there is a small possibility that the fitness level of the older adult group was lower than that of the young and middle‐aged groups: we did not attempt to compare fitness levels between the groups. Third, in the present study, some participants were active smokers, and BP response is affected by smoking (Papathanasiou et al., 2007). However, no significant differences in the proportion of smokers in the three age groups suggests that the effect of smoking on the BP response would be minimal in the present study.

Despite these limitations, this study has several strengths. First, while nearly all the studies investigating the effect of aging on pressor response to exercise have compared young and old age groups (Markel et al., 2003; Milia et al., 2015; Sidhu et al., 2015; Trinity et al., 2018), the present study compared three age ranges, which lent additional insight into the potential age threshold when the BP response to ischemic exercise starts becoming exaggerated. Second, by performing an additional multivariate analysis, we found that aging was a significant independent determinant of the BP response to ischemic exercise.

## 5 CONCLUSION

The present study demonstrates that aging enhances the pressor response to ischemic rhythmic exercise.

## DISCLOSURES

The authors declare no conflict of interest, financial, or otherwise.

## AUTHOR CONTRIBUTIONS

Daisuke Hasegawa and Norio Hotta conceived and designed the research; Daisuke Hasegawa, Amane Hori, Yukiko Okamura, Reizo Baba, Kenichi Suijo, Koji Kitatsuji, and Norio Hotta performed experiments; Daisuke Hasegawa, Amane Hori, Kenichi Suijo, Jun Sugawara, and Norio Hotta analyzed data; Daisuke Hasegawa, Amane Hori, Yukiko Okamura, Reizo Baba, Kenichi Suijo, Masaki Mizuno, Jun Sugawara, Koji Kitatsuji, Hisayoshi Ogata, Kaoru Toda, and Norio Hotta interpreted results of experiments; Daisuke Hasegawa, Amane Hori, and Norio Hotta prepared the figures; Daisuke Hasegawa, Amane Hori, and Norio Hotta drafted the manuscript; Daisuke Hasegawa, Amane Hori, Reizo Baba, Jun Sugawara, Masaki Mizuno, and Norio Hotta edited and revised the manuscript; Daisuke Hasegawa, Amane Hori, Yukiko Okamura, Reizo Baba, Kenichi Suijo, Masaki Mizuno, Jun Sugawara, Koji Kitatsuji, Hisayoshi Ogata, Kaoru Toda, and Norio Hotta approved the final version of the manuscript.
